# USP21 modulates Goosecoid function through deubiquitination

**DOI:** 10.1042/BSR20182148

**Published:** 2019-07-10

**Authors:** Fuwei Liu, Qian Fu, Yunpeng Li, Kai Zhang, Mingyue Tang, Wei Jiang, Bin Bo, Yajun Cui, Liang Kong

**Affiliations:** 1 State Key Laboratory of Military Stomatology & National Clinical Research Center for Oral Disease & Shaanxi Key Laboratory of Oral Diseases, Department of Oral and Maxillofacial Surgery, School of Stomatology, The Fourth Military Medical University, Xi’an 710032, People’s Republic of China; 2 Orthopedic Research Laboratory, Boston Children’s Hospital, 300 Longwood AVE, Boston, MA 02115, USA

**Keywords:** Deubiquitination, Goosecoid, Sox6 reporter gene system, USP21

## Abstract

The homeobox gene Goosecoid (*GSC*), which is known to regulate craniofacial development, is activated by mono-ubiquitination; however, the deubiquitylase responsible for GSC deubiquitination and inhibition has yet to be identified. In the present study, we constructed the recombinant plasmid pFlag-CMV-2-GSC and the SRY (sex-determining region Y)-box 6 (*Sox6*) reporter gene system to identify deubiquitylases that regulate GSC expression. We demonstrate that the ubiquitin carboxyl-terminal hydrolase 21 (USP21) regulates the deubiquitination of GSC negatively, as demonstrated by its inhibition of *Sox6* reporter gene transcription. USP21 interacted with GSC to promote GSC deubiquitination while having no effect on GSC protein stability. Cell viability, migration, and function in ATDC5 cells were probably influenced by USP21 through GSC. These findings suggest that USP21 modulates GSC function through deubiquitination.

## Introduction

Goosecoid (*GSC*), a homeobox gene expressed mainly in the craniofacial region, plays an important role in regulating craniofacial development, which acts downstream of regulatory networks specifying neural crest cell fate and determines mesoderm cell lineages in mammals [1,2]. Targeted mutation of the murine *Gsc* gene resulted in craniofacial defects and neonatal death [3]; in humans, analogous mutations cause a syndrome characterised by short stature, auditory-canal atresia, mandibular hypoplasia, and skeletal abnormalities [2].

GSC is a transcriptional activator of the cartilage regulatory protein SRY (sex-determining region Y)-box (SOX)6. Both WW domain-containing 2 (WWP2, UNIPROT: O00308) and cadherin 1 (CDH1, UNIPROT: Q9UM11)-dependent anaphase–promoting complex E3 ubiquitin ligases interact with and induce mono-ubiquitination of GSC, which is required for its transcriptional activation [4]. Simultaneous deletion of CDH1 and WWP2 caused more severe craniofacial defects compared with single gene deletion, suggesting a synergistic enhancement of GSC activity by these two factors. Mice deficient in WWP2 develop craniofacial malformations similar to those lacking GSC [3,5]. Accordingly, neural crest-specific *Cdh1* knockout mice exhibit a domed skull, short snout, and a twisted nasal bone [6]. These results highlight the importance of ubiquitination in the activity of GSC in craniofacial development. However, the detailed mechanisms underlying GSC regulation and activity have not been elucidated.

To this end, in the present study, we used a *Sox6* reporter gene system to identify deubiquitylases (DUBs) that specifically regulate GSC stability. Of the 41 DUBs that were screened in human embryonic kidney (HEK) 293T cells, ubiquitin carboxyl-terminal hydrolase 21 (USP21, UNIPROT: Q9UK80) was identified as a regulator of GSC stability.

## Materials and methods

### Cell culture and reagents

HEK 293T cells were cultured in Dulbecco’s modified Eagle’s medium (DMEM) supplemented with 10% fetal bovine serum (FBS; Hyclone, Logan, UT, U.S.A.) at 37°C under 5% CO_2_. ATDC5 cells, a murine chondrocytic cell line, were cultured in DMEM/F12 medium (Hyclone) containing 1 mM phosphorus, 0.25 mM glycine, and 0.15 mM proline. In addition, the following reagents were used in the study: 2OD primers (Sangon Biotech, Shanghai, China); EcoRI and BglII restriction enzymes (Thermo Fisher Scientific, Waltham, MA, U.S.A.); T4 ligase (Thermo Fisher Scientific); DL-2000 DNA Marker (Thermo Fisher Scientific); high-glucose DMEM (Hyclone); trypsin (Hyclone); cycloheximide (CHX) (Sigma–Aldrich, St. Louis, MO, U.S.A.); anti-Myc (M4439), anti-Flag (F3165), anti-HA (H9658), anti-Col II (MAB8887), anti-heat shock protein (HSP)90 (SAB1305541) primary antibodies (Sigma–Aldrich); and anti- mouse IgG (A9044, Sigma–Aldrich).

### Construction of recombinant eukaryotic expression vector

RNA was extracted from HEK 293T cells and reverse transcribed to cDNA, which was used as a template to amplify the *GSC* and all the *DUBs* gene. After PCR amplification, the products were identified by agarose gel electrophoresis and then purified. The amplicon and pFlag-CMV-2 vector were digested with restriction enzymes, identified and purified by agarose gel electrophoresis. The above enzymatic digestion products were connected using T4 DNA ligase, to achieve that the target genes were inserted into the pFlag-CMV-2 vector. The recombinant vectors were transferred into competent *Escherichia coli* DH5α cells. Positive clones were confirmed by sequencing. Thus, stable expression of GSC and the 41 DUBs vectors were achieved. Both pFlag-CMV-2 and *E. coli* DH5α cells were gifts from Professor Lingqiang Zhang (Military Medical Science Academy of the PLA, Beijing, China). The *GSC* primers used in the present study were 5′-TACCGGAATTCAATGCCCGCCAGCATGTTCAGCATCGA-3′ (forward) and 5′-TCGGAAGATCTCTGTGCAAGTCCTTCGAGTTAGG-3′ (reverse).

### Cell transfection

Cells were transfected with plasmids using Lipofectamine 2000 (Invitrogen, Carlsbad, CA, U.S.A.) according to the manufacturer’s instructions. HEK 293T cells were seeded (5 × 10^4^ cells/well) in 24-well plates and incubated overnight at 37°C in a 5% CO_2_ incubator. Before transfection, the medium was replaced with 500 μl of fresh DMEM without FBS or antibiotics. Upon reaching 80% confluence, cells were transfected by adding 1 μg of pFlag-CMV-2 vector dissolved in 50 μl of serum-free medium to each well, followed by 3 μg of Lipofectamine 2000 in 50 μl of serum-free medium for 5 min; 100-μl solutions containing Lipofectamine-pFlag-CMV-2 complexes were then added to the cells, followed by incubation for 6 h in a humidified incubator at 37°C under 5% CO_2_. The medium was replaced with fresh medium containing 10% FBS and antibiotics, and the cells were cultured for another 24 h. Images of 293T cells transfected with plasmids were taken using optical microscope and fluorescence microscope to determine the cell transfection efficiency. Experiments were performed at 24 or 48 h after transfection when the transfection efficiency was higher than 80%. The same procedures were used to transfect ATDC5 cells.

### Dual-luciferase reporter assay

The luciferase assay was performed using a Dual-Luciferase Reporter Assay kit (Promega, Madison, WI, U.S.A.), according to the manufacturer’s instructions. HEK 293T cells grown in a 24-well plate were transiently co-transfected with firefly luciferase reporter vectors (*Sox6*-pro238 and -pro273 constructs and GSC-encoding plasmid), effector vectors, and the empty luciferase reporter construct mixed with Lipofectamine 2000. At 48 h after transfection, cells were harvested in passive lysis buffer and firefly and *Renilla* luciferase activities were measured separately using a fluorescence spectrophotometer. The relative transcriptional activity was normalised to the value corresponding to the vehicle control.

### Western blotting

Proteins were extracted from stably transfected cells lysed in radioimmunoprecipitation assay lysis buffer, and the protein concentration was determined using the Bicinchoninic Acid (BCA) Protein Quantification kit (Vazyme, Piscataway, NJ, U.S.A.). After heat denaturation in 5% sodium dodecyl sulphate polyacrylamide gel electrophoresis (SDS/PAGE) sample loading buffer, protein samples were resolved by SDS/PAGE and transferred to a polyvinylidene difluoride membrane. The membrane was probed with anti-Myc, -Flag, -HSP90 and HA antibodies (all at 1:1000 from Sigma–Aldrich). Horseradish peroxidase-conjugated anti-rabbit/mouse IgG (1:1000; Sigma–Aldrich) was used as a secondary antibody. Immunoreactivity was detected using enhanced chemiluminescence Western blotting substrate (Beyotime Institute of Biotechnology, Shanghai, China), and the results were analysed using an automatic gel imaging system (Kodak Digital, Rochester, NY, U.S.A.).

### Co-immunoprecipitation assay

HEK 293T cells were co-transfected with pFlag-CMV-2 and GSC or USP21 plasmids. After 48 h, the cells were harvested and washed twice with chilled phosphate-buffered saline, and then lysed in HEPES lysis buffer composed of 20  mM HEPES (pH 7.2), 50  mM NaCl, 1  mM NaF, 0.5% Triton X-100, and protease inhibitor cocktail (Sigma–Aldrich). Total protein was quantified using a BCA Protein Assay kit, and diluted to 1 mg/ml with chilled wash buffer containing protease inhibitors. A 40-μl volume was removed as input and stored at −20°C. The remaining protein was combined with the mouse anti-Flag and anti-Myc antibody (1 μg; Sigma–Aldrich) with slow rotation for 3 h at 4°C. Protein agarose beads were washed twice with cold wash buffer, combined with the protein, and incubated for 8 h at 4°C with low-speed rotation. The protein mixture was washed three times with 800 μl of chilled Tris-buffered saline (containing a protease inhibitor), and 40 μl was stored at −20°C as the immunoprecipitated fraction until use. Protein mixtures, including the input, were diluted with wash buffer. After heat denaturation in 5% SDS–PAGE sample loading buffer, samples subjected to SDS/PAGE and analysed by Western blotting.

### *In vitro* GSC mono-ubiquitylation assay

For the *in vitro* GSC mono-ubiquitylation assay, HEK 293T cells were transfected with GSC, WWP2, USP21, and HA-Ub-K0 plasmids; 48 h later, the cells were subjected to Co-immunoprecipitation (co-IP). To determine the level of ubiquitination of the proteins, biotinylated ubiquitin was detected by Western blotting as described above. HA-Ub-K0, which comprises a protein tag ‘HA’ and ubiquitin without lysine, was a gift from Professor Lingqiang Zhang (Military Medical Science Academy of the PLA, Beijing, China). WWP2 also was a gift from Professor Zhang.

### Protein half-life assay

For the GSC half-life assay, HEK 293T cells were grown in 12-well plates until 60% confluence and then transfected with plasmids encoding GSC and USP21; 48 h later, the cells were treated with the protein synthesis inhibitor CHX (40 ng/μl; Sigma–Aldrich) and harvested at 0, 2, 6, 8, and 12 h. Lysates were subjected to Western blotting, and the half-life was calculated.

### MTT assay

Cell growth rates were determined by the 3-(4,5-dimethythiazol-2-yl)-2,5-diphenyl tetrazolium bromide (MTT, Sigma, U.S.A.) assay according to the manufacturer’s protocol (Vybrant, Molecular Probes, Leiden, Netherlands). Cells (1 × 10^4^) were plated on 96-well micro-plates. Absorbance was read at 570 nm in triplicate.

### Trypan blue staining

Cellular viability was determined by counting with a Neubauer counting chamber (Marienfeld) using trypan blue (BBI, 0.4% in PBS) discrimination. A single-cell suspension was prepared and diluted to the appropriate concentration (10^6^ cells/ml). The cell suspension was mixed with 0.4% trypan blue solutions at 9:1. Then, live and dead cells were counted under a microscope in 3 min. Percentage of living cells = total number of living cells /(total number of living cells + dead cells) ×100%. The relative percentage against the control was calculated.

### Colony formation assay

ATDC5 and transfected cells were seeded into 24-well culture plate at 200 cells/dish for colony formation. The dishes were precoated with 2% low-melting-point agarose. After culture for 14 days at 37°C, cells were fixed with 4% paraformaldehyde and stained with 0.1% crystal violet (Sangon Biotech, China). Visible colonies were counted under an optical microscope. The relative percentage against the control was calculated.

### Cell scratch assay

As an *in vitro* model of wound healing, a scratch test was also conducted to evaluate the ability of USP21 to influence the spreading and migration of chondrocytes. Briefly, ATDC5 cells (5.0 × 10^3^ cells/cm^2^) were seeded in 6-well plates, and when a confluent monolayer was obtained, a linear scratch was generated using a sterile pipette tip. ATDC5 cells transfected with USP21 plasmids were regarded as the experiment group. Empty plasmid was added to the negative control wells. Samples were run in quadruplicate. Plates were incubated at 37°C under 5% CO_2_ and photographs were taken at a 4× magnification on days 1, 2, and 3.

### Toluidine blue staining

To analyse the matrix secretion of chondrocytes, toluidine blue staining assay was conducted. Cells were prepared in cell climbing sheets until the cells reached 80–90% confluence. After washed three times with PBS, cells were fixed with 4% paraformaldehyde for 15 min, and then dyed with 0.5% toluene blue solution at 50°C for 30 min. Cell climbing sheets were rinsed with distilled water, dried, and then photographed.

### Analysis of Col II mRNA expression by qPCR assay and protein level by Western blot assay

To simulate the state of mature chondrocytes, we conducted cartilage induction in ATDC5 cells. Cells were plated on 12-well plates with a concentration of 4×10^5^/ml. When the cells reached 90% confluence, fresh DMEM/F12 without FBS was added for incubation overnight. The medium was then changed to complete DMEM/F12 with ITS inducer (insulin-transferrin-sodium selenite), and the induction of differentiation was marked as day 0. After induction for 14 days, cells were collected to extract total RNA using Trizol reagent (Invitrogen) in accordance with the manufacturer’s instructions. The quantity and quality of RNA were determined using a BioAnalyzer 2100 (Agilent, Santa Clara, CA, U.S.A.) prior to further processing. The mRNA expression of Col II was detected by qPCR (real-time quantitative PCR), and their protein expression was determined by Western blot.

### Statistical analysis

All assays were performed three times. Data are expressed as the mean ± SD, and means were compared using Student’s *t* test or two-way analysis of variance. A two-tailed *P*<0.05 was considered statistically significant.

## Results

### Construction of GSC-Sox6 reporter gene system

**Figure 1:**
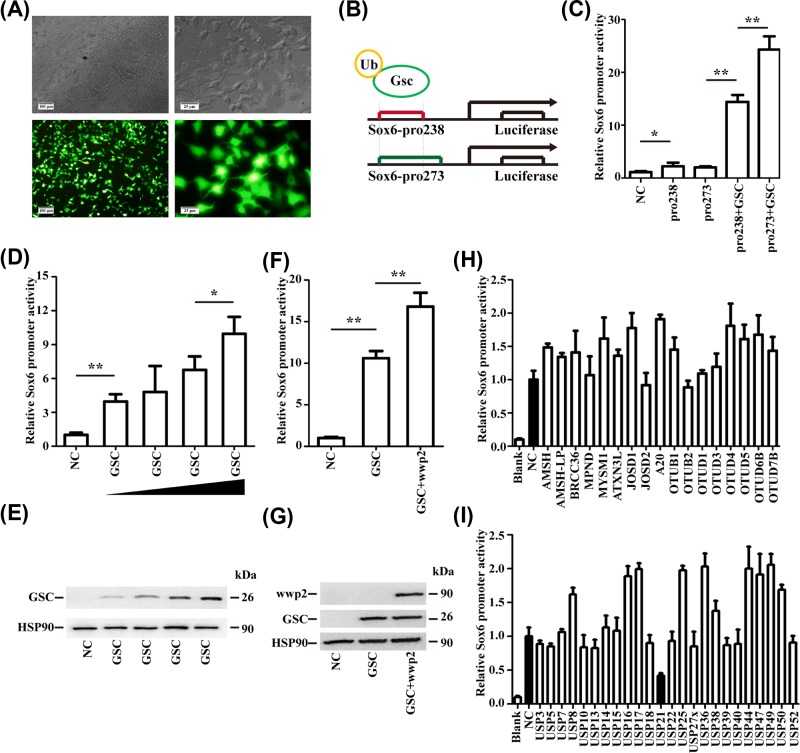
Establishment of the GSC-Sox6 reporter gene system and screen for the DUB of GSC (**A**) Micrograph of 293T cells transfected with the plasmid under optical microscope and fluorescence microscope. (scale of left side = 100 μm, scale of left side = 25 μm). (**B**) Maps of the two Sox6 reporter gene constructs (Sox6-pro238 and -pro273). (**C**) Activity of Sox6-pro238 and -pro273 upon co-transfection of GSC plasmid. Sox6-pro273 was selected for the reporter gene screening system. GSC, Goosecoid; Sox-6, SRY (sex-determining region Y)-box 6. (**D,E**) Effect of GSC expression level on Sox6-pro273 activity in HEK 293T cells. (**F,G**) Effect of GSC and WWP2 co-expression on Sox6-pro273 activity. The activity of the Sox6-pro273 reporter gene was positively correlated with GSC expression level and mono-ubiquitination level. (**H,I**) Screen for the DUB of GSC. HEK 293T cells expressing the GSC-Sox6 reporter and indicated proteins; firefly luciferase activity was normalised to that of *Renilla* luciferase and then to that of the control vector. USP21 decreased GSC-Sox6 reporter gene activity. DUB, deubiquitylase; GSC, Goosecoid; HSP-90, heat shock protein 90; Sox-6, SRY (sex-determining region Y)-box 6; USP21, ubiquitin-specific protease 21; Wwp2, WW domain-containing 2. *P<0.05, **P<0.01.

A GSC eukaryotic expression vector was constructed and used to establish a GSC-*Sox6* reporter gene system using *Sox6*-pro238 and -pro273 constructs (Figure 1B). The transfection efficiency of the two reporter plasmids in HEK 293T cells was >80% (Figure 1A). The activity of *Sox6*-pro273 increased ∼12-fold as compared with the 8-fold increase in *Sox6*-pro238 activity in the presence of GSC expression. Because *Sox6*-pro273 was more responsive to GSC, we used this vector for the reporter gene screening system (Figure 1C).

### Evaluation of GSC-Sox6 reporter gene system efficiency

Cells were transfected with different concentrations of the GSC vector. *Sox6* reporter gene activity was positively correlated with the level of GSC (Figure 1D,E). *Sox6*-pro273 activity in cells transfected with GSC was ∼11 times higher than that in control cells, whereas co-transfection of GSC and WWP2 increased the reporter gene activity by 17-fold (*P*<0.01, Figure 1F,G). Moreover, GSC ubiquitination was associated with an increase in *Sox6* reporter activity. These results confirmed that the GSC-*Sox6* reporter gene system could be used to screen for DUBs of GSC: when GSC expression decreased, the activity of the luciferase gene fused with Sox6-pro273 decreased accordingly, and hence the decrease in GSC expression could be quantified by measuring the luciferase activity.

### USP21 is the DUB of GSC

Forty-one mammalian DUBs were screened using the *Sox6*-pro273 reporter construct. We found that the relative expression of the *Sox6* reporter gene fluctuated between 0.8 and 2 after transfection of different ubiquitination enzymes. Co-expression of USP21 and GSC clearly decreased the luciferase activity, with a relative activity of ∼0.4 (Figure 1H,I). It is suggested that USP21 is the DUB of GSC, suggesting that measurement of GSC ubiquitination and the effect of ubiquitination on *Sox6* expression required to be tested. In addition, other DUBs did not affect the reporter gene activity, although some increased the activity, possibly because of GSC multi-ubiquitination.

### USP21 regulates GSC activity negatively

**Figure 2:**
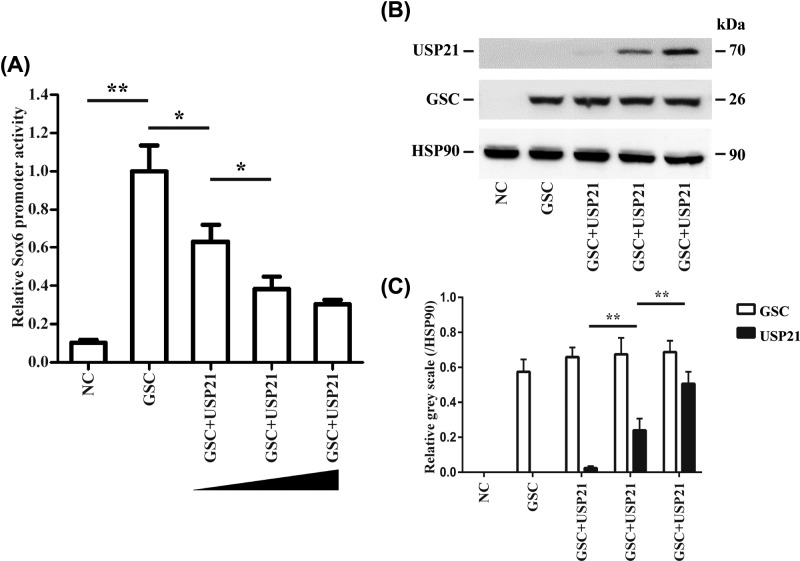
Effect of USP21 on GSC-*Sox6* reporter gene activity upon USP21 overexpression (**A**) *Sox6*-pro273 activity was negatively correlated with USP21 expression level. (**B**) Expression level of GSC upon USP21 gradient overexpression in HEK 293T cells. (**C**) Intensities and statistical analysis of the protein expression of GSC and USP21. Group classification: NC group indicates the negative control, ATDC5 cells transfected with empty plasmid only. GSC group indicates ATDC5 cells transfected with the GSC plasmid. GSC+USP21 group indicates ATDC5 cells transfected with the GSC and USP21 plasmid. GSC, Goosecoid; HSP-90, heat shock protein 90; Sox-6, SRY (sex-determining region Y)-box 6; USP21, ubiquitin-specific protease 21; *P<0.05, **P<0.01.

After transfection with GSC and gradient USP21 in HEK 293T cells, *Sox6*-pro273 activity was found to decrease as a function of USP21 expression level (Figure 2A). Western blot also confirmed that USP21 influenced the activity of GSC-*Sox6* reporter gene as the protein level of GSC was consistent (Figure 2B,C).

### USP21 interacts with GSC

**Figure 3:**
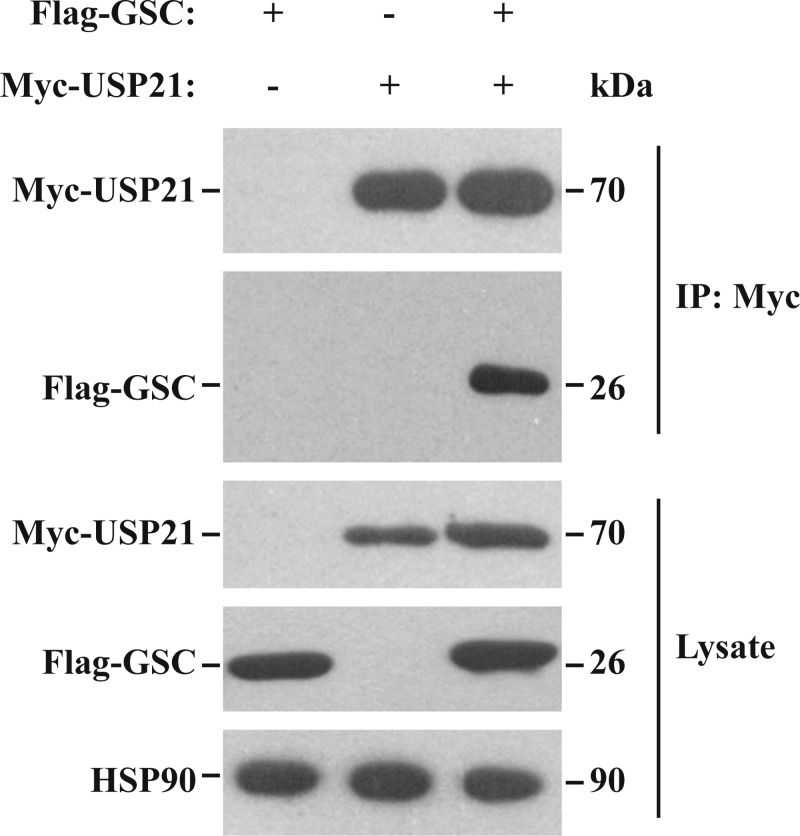
Interaction of USP21 and GSC. The interaction of Flag-tagged GSC (Flag-GSC) and Myc-tagged USP21 (Myc-USP21) was analysed by co-immunoprecipitation Western blotting was performed with an anti-flag antibody for Flag-GSC, anti-Myc antibody for Myc-USP21, and an anti-HSP90 antibody. HEK 293T cells were transfected with a plasmid encoding Myc-USP21 and one encoding Flag-GSC or empty vector. Myc-USP21 was immunoprecipitated with anti-Myc antibody and immunoblotted with anti-Flag antibody to detect GSC. Flag-GSC was combined with Myc-USP21 and the proteins co-precipitated, indicating that USP21 interacts with GSC. HSP90 served as a loading control. Data are representative of at least three independent experiments. GSC, Goosecoid; HSP-90, heat shock protein 90; USP21, ubiquitin-specific protease 21.

To determine whether USP21 interacts with GSC, HEK 293T cells were co-transfected with expression vectors encoding Flag epitope-tagged GSC (Flag-GSC) and Myc-tagged USP21 (Myc-USP21), followed by reciprocal co-IP. Flag-GSC was pulled down together with Myc-USP21 (Figure 3), indicating that that the two proteins interact.

### USP21 promotes GSC deubiquitination

**Figure 4:**
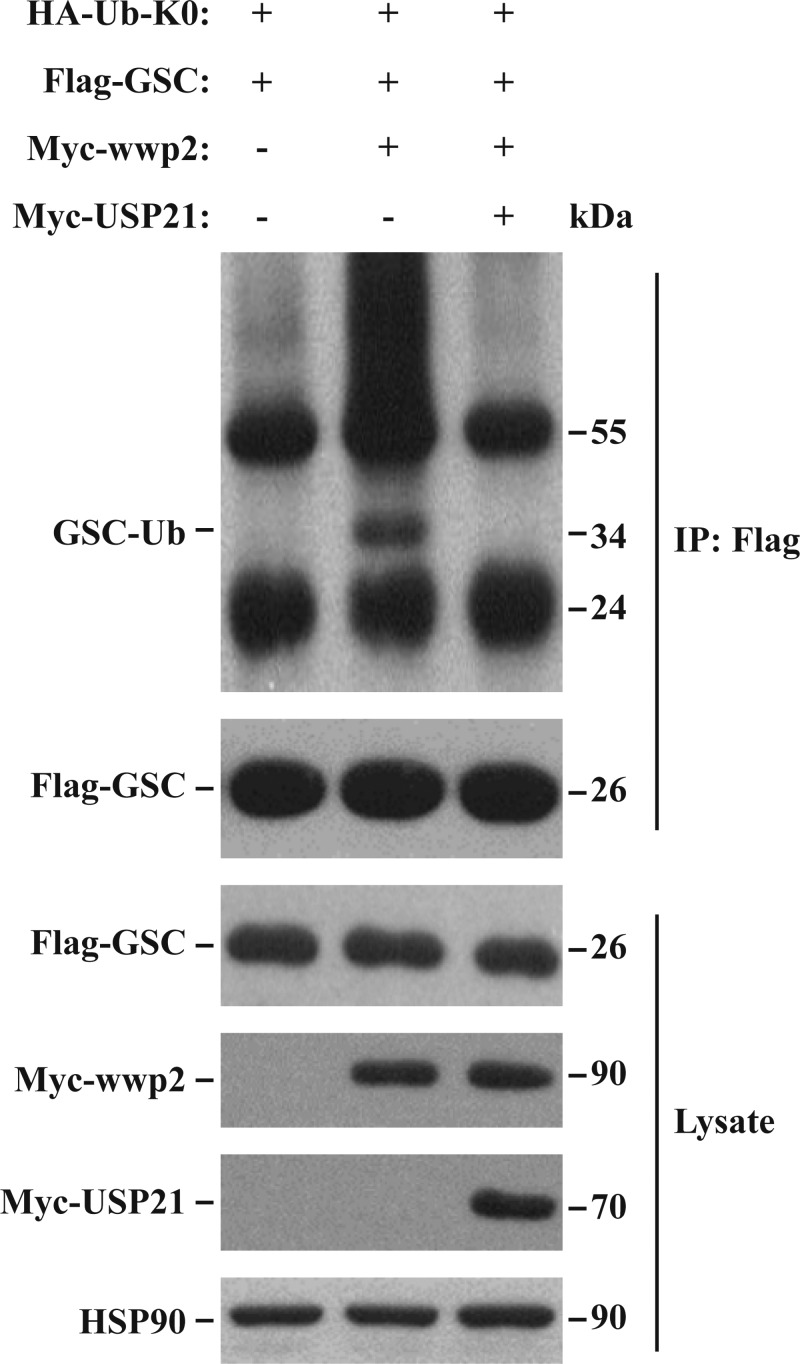
Effect of USP21 on GSC ubiquitylation. HEK 293T cells were co-transfected with HA-Ub-K0, Flag-GSC, Myc-USP21, or Myc-WWP2 Western blotting was performed with an anti-flag antibody for Flag-GSC, anti-Myc antibody for Myc-USP21 and Myc-WWP2, anti-HA antibody for GSC-Ub, and anti-HSP90 antibody for HSP90. Flag-GSC was immunoprecipitated with anti-Flag antibody. WWP2 overexpression increased GSC mono-ubiquitination, and USP21 overexpression decreased GSC mono-ubiquitination, indicating that USP21 acts as a DUB of GSC. HSP90 served as a loading control. Data are representative of at least three independent experiments. DUB, deubiquitylase; GSC, Goosecoid; HSP-90, heat shock protein 90; HA, HA tag; Myc, Myc tag; Ub, ubiquitin; USP21, ubiquitin-specific protease 21; Wwp2, WW domain-containing 2.

To determine whether USP21 is a DUB of GSC, HEK 293T cells were co-transfected with Flag-GSC and HA-ubiquitin-K0 (HA-Ub-K0), with or without Myc-USP21 and Myc-WWP2; after 2 days, immunoprecipitation was performed using an anti-Flag antibody, and immunoblotting was performed with anti-HA and anti-Flag antibodies. Although there was no evidence of interaction between Flag-GSC and HA-Ub-K0 (Figure 4), GSC was mono-ubiquitinated in the presence of Myc-WWP2. This was abrogated upon addition of Myc-USP21, indicating that USP21 is a DUB of GSC.

### GSC is a substrate of USP21

**Figure 5:**
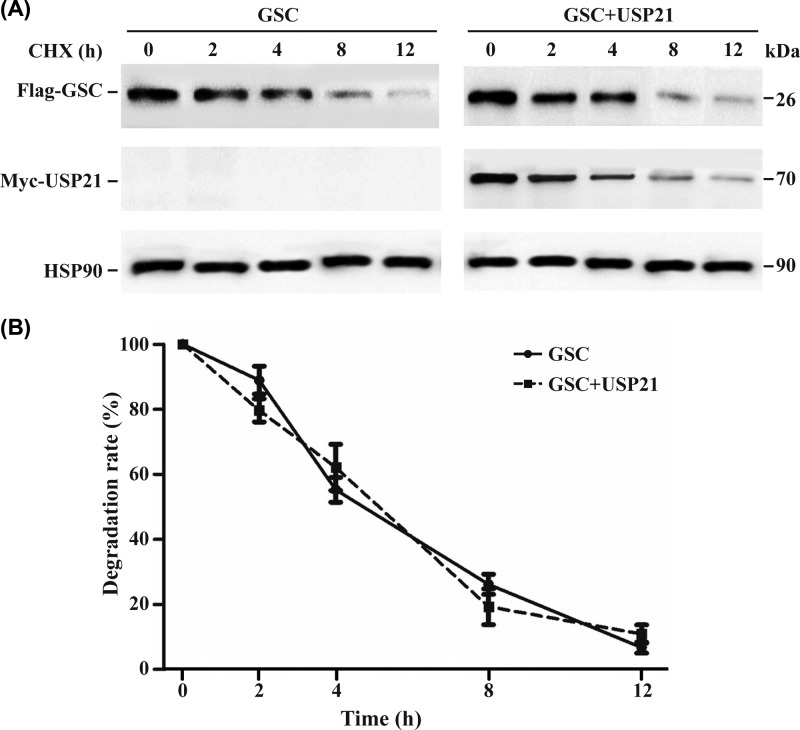
Analysis of GSC half-life. HEK 293T cells were transfected with Flag-GSC alone or with Myc-USP21 Protein levels were analysed 0, 2, 6, 8, and 12 h after treatment with CHX. (**A**) GSC level was quantified relative to that of HSP90 by Western blotting. USP21 acted as a mono-DUB of GSC. (**B**) The degradation rate as a graph with statistical evaluation is shown. CHX, cycloheximide; DUB, deubiquitylase; GSC, Goosecoid; HA, HA tag; HSP-90, heat shock protein 90; Myc, Myc tag; USP21, ubiquitin-specific protease 21.

To confirm that USP21 deubiquitinates GSC, we inhibited protein synthesis by treating cells with CHX and examining lysates at specific times to measure the half-life of GSC. Cells transfected with Flag-GSC only or both Flag-GSC and USP21 showed similar decreases in GSC and USP21 over time (Figure 5A,B), indicating that USP21 does not affect the half-life of GSC. We speculated that USP21 is a mono-DUB of GSC.

### USP21 influences the function of GSC in ATDC5 cells

To determine whether USP21 influences the function of GSC in chondrocytes, we performed MTT, trypan blue staining, and colony formation assays to determine cell viability, cell scratch assay to detect cell migratory ability, toluidine blue staining to analyse the matrix secretion, and qPCR and Western blot assays to detect cartilage differentiation-related molecules, respectively. ATDC5 cells were prepared into four groups: transfected with empty plasmid (negative control), both GSC plus USP21 plasmids, GSC plasmid, or USP21 plasmid. The negative control group was included to eliminate the interference of blank plasmids.

**Figure 6:**
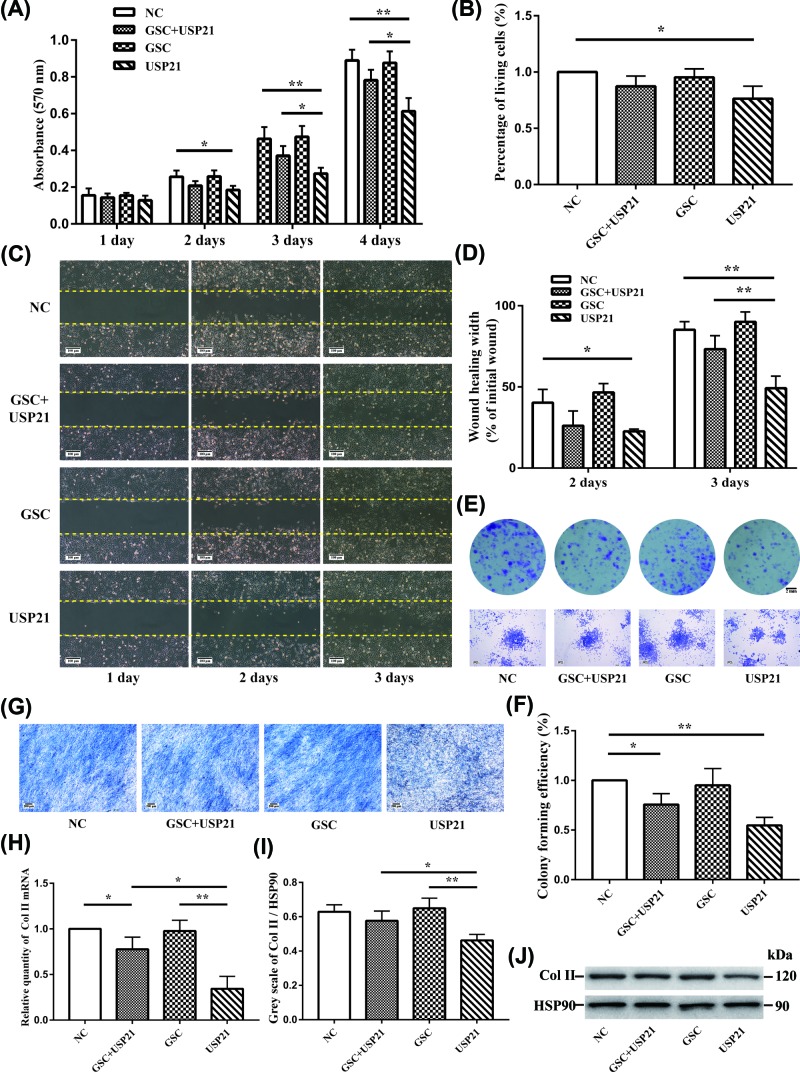
Effect of USP21 on GSC in ATDC5 cells (**A**) The signal intensity of the viability of the ATDC5 cells in 1–4 days was determined by the MTT assay. The absorbance of the USP21 group showed an obvious decline compared with that of the other groups. (**B**) Cellular viability was determined by counting with a Neubauer counting chamber using Trypan blue discrimination in the different transfected ATDC5 groups. (**C,D**) Effect of USP21 in scratch wound healing model using ATDC5. The migration rate of cells into the scratched site was evaluated in the different transfected groups at days 1, 2, and 3 after incubation. Photographs were taken at a 4× magnification (C, scale = 100 μm); Graph of wound healing rate with statistical evaluation (D). (**E,F**) Effect of USP21 and GSC on survival of ATDC5 cells determined with the colony formation assay. Photographs were taken under optical microscope (E, scale = 200 μm); the number of clones were statistical analysed (F). (**G**) Effect of USP21 and GSC on the matrix secretion of ATDC5 cells determined with the toluidine blue staining assay (scale = 200 μm). (**H–J**) Effect of USP21 on GSC target gene Col II mRNA level and protein level upon USP21 or/and GSC overexpression. After cartilage induction for 14 days in ATDC5 cells, the mRNA expression of Col II were quantified with qPCR assay (H), the protein expression of Col II was quantified with Western blot assay (I,J). Intensities and statistical analysis of the protein expression of Col II (J). Group classification: NC group indicates the negative control, ATDC5 cells transfected with empty plasmid only. GSC+USP21 group indicates ATDC5 cells transfected with the GSC and USP21 plasmids. GSC group indicates ATDC5 cells transfected with the GSC plasmid. USP21 group indicates ATDC5 cells transfected with the USP21 plasmid. Col, collagen; GSC, Goosecoid; HSP-90, heat shock protein 90; MTT, 3-(4,5-dimethythiazol-2-yl)-2,5-diphenyl tetrazolium bromide; qPCR, real-time quantitative PCR; USP21, ubiquitin-specific protease 21. *P<0.05, **P<0.01.

According to the MTT results (Figure 6A), there was an obvious decline in the viability of ATDC5 cells when USP21 was transfected compared with the other three groups. After adding GSC, the cell viability increased obviously, which was only slightly less than that of NC. For the cell scratch assay, the migratory ability of ATDC5 transfected with USP21 showed a significant delay, an improvement after adding GSC (Figure 6B,C). For the trypan blue staining, the percentage of living cells in the USP21 group was lower than that in NC group, but not significantly (*P*<0.05), suggesting that USP21 partly affected cell apoptosis (Figure 6D). For the colony formation assay, USP21 decreased the colony formation efficiency significantly (*P*<0.01), and that in GSC+USP21 decreased slightly compared with NC group (*P*<0.05) (Figure 6E,F). For the toluidine blue staining, USP21 decreased the matrix secretion of ATDC5 significantly and no obvious difference exists in the other three groups (Figure 6G). Additionally, the cartilage differentiation-related molecules Col II were detected at the mRNA level (Figure 6H) and protein level (Figure 6I,J) after cartilage induction for 14 days, to analyse the effect of USP21 on cartilage function in ATDC5 cells. Results showed that USP21 overexpression decreased Col II expression, and there was a distinct rebound in GSC+USP21 overexpression group.

## Discussion

GSC is a paired-like homeodomain-containing transcription factor expressed in early gastrulating embryos [7] that regulates the transcription of its target genes by binding to paired TAAT-binding sites or via recruitment by the transcription factor, Forkhead box h1 [8,9]. GSC is enriched in cartilage, and plays an important role in neural crest cell migration and differentiation during craniofacial development [10–13].

Posttranslational modifications regulate protein activity, interactions, subcellular localisation, and stability [14]. GSC is subject to various types of posttranslational modifications. For instance, in mammalian cells, the transcriptional suppressor function of GSC is regulated via small ubiquitin-like modifier (SUMO) by protein inhibitor of activated STAT [8,13,15]. Mono-ubiquitination is a posttranslational modification mediated by ubiquitin ligases that regulates protein functions and is critical to craniofacial development [5,6]. DUBs mediate the removal and processing of ubiquitin [16].

In the present study, we identified USP21 as a DUB that regulates the functional status of GSC but not the protein level of GSC via a non-proteolytic mechanism. USP21 was found to interact with GSC, similar to other ubiquitin-specific proteases [17–19]. Co-expression of USP21 and GSC in HEK 293T cells did not alter GSC protein levels, suggesting that proteasome-mediated degradation was not induced [20,21]. USP21 contains highly conserved His and Cys domains characteristic of the ubiquitin-specific C19 cysteine protease family that disassemble various forms of polymeric ubiquitin or ubiquitin-like protein complexes [17,18]. Human USP21 comprises 565 amino acids and adopts the common three-subdomain architecture of USPs [20,22]. The covalently attached proximal moiety of linear di-ubiquitin binds to the S1 site of USP. USP21 also has a small S2 binding site consisting of Arg 441, Gln 442, Lys 443, and Thr 444 that does not contribute to ubiquitin chain hydrolysis, but participates in SUMO modification [20,21].

USP21 acts as a negative regulator in antiviral responses through its ability to bind to and deubiquitinate RIG-I. USP21 overexpression was reported to inhibit RNA virus-induced RIG-I polyubiquitination and interferon signalling [23]. USP21 also negatively regulates anti-DNA virus immunity by deubiquitinating STING [24]. USP21 was shown to modulate the transcription of nuclear factor-κB p65 by deubiquitinating and stabilising interleukin-33 in the nucleus [25], and interacts with both nuclear and cytoplasmic proteins including GATA3, receptor-interacting serine/threonine-protein kinase 1, and Nanog [14,19,26,27]. A USP21 short variant lacking a nuclear export signal was observed to be primarily localised in the nucleus, suggesting a unique nuclear function [28,29]. Furthermore, USP21 has been shown to associate with both centrosomes and microtubules to regulate cell motility in three-dimensional matrices [29–33], and promotes cell proliferation and metastasis in kinds of cancer [34–37]. Our results suggest that USP21 probably influence the function of GSC and further decrease the viability, migration, and function of chondrocytes, verifying the results of previous studies that indicated [5,13]. Thus, USP21 and GSC may cooperate to influence the cartilage function and regulate the cytoskeleton during cell migration and differentiation [38–40].

Linear deubiquitination plays an important role in angiogenesis and craniofacial and neural development [41]. Spleens from *Usp21*-deficient mice were larger than those from wild-type littermates [23]; however, another study showed that *Usp21* mutants were viable and fertile, with no obvious morphological abnormalities [42]. We believe that further specific examinations of maxillofacial surgery are needed, as they may reveal some detailed abnormalities. In the present study, we demonstrated, for the first time, that USP21 could regulate GSC specifically in craniofacial tissues by reversing its mono-ubiquitination.

In summary, we identified USP21 as a specific DUB for the transcription factor GSC, which is presumed to play a critical role in regulating the function of GSC by removing its mono-ubiquitin, thereby modulating the expression of cartilage-related factors. Meanwhile, the preliminary experiments in chondrocytes indicated that USP21 probably influence cartilage function through GSC. These findings may provide a theoretical basis for explaining some diseases caused by cartilage abnormalities. However, additional work is needed to determine whether GSC is ubiquitinated at Lys63 rather than Lys48 of ubiquitin using C211S as a negative control. In addition, investigations using primary cells of craniofacial tissues and transgenic animal models are necessary to confirm our findings. And the direct evidences for the relationship between USP21 and craniofacial development are needed, which will be confirmed in our further study.

## Supporting information

**Supplementary Figure S1:** 

**Supplementary Table S1:** Information about the Antibodies.

## Abbreviations

BCA: bicinchoninic acid
CHX: cycloheximide
Co-IP: co-immunoprecipitation
DMEM: Dulbecco’s modified Eagle’s medium
DUB: deubiquitylases
FBS: fetal bovine serum
Flag-GSC: flag epitope-tagged GSC
GSC: Goosecoid
HEK: human embryonic kidney
qPCR: real-time quantitative PCR
SDS/PAGE: sodium dodecyl sulphate polyacrylamide gel electrophoresis
Sox6: SRY (sex-determining region Y)-box 6
SUMO: small ubiquitin-like modifier
USP21: ubiquitin-specific peptidase (USP)21

## References

[1] Niehrs C., Keller R., Cho K.W. and De Robertis E.M. (1993) The homeobox gene goosecoid controls cell migration in Xenopus embryos. Cell 72, 491–503 10.1016/0092-8674(93)90069-3 8095000

[2] Parry D.A., Logan C.V., Stegmann A.P., Abdelhamed Z.A., Calder A., Khan S. (2013) SAMS, a syndrome of short stature, auditory-canal atresia, mandibular hypoplasia, and skeletal abnormalities is a unique neurocristopathy caused by mutations in Goosecoid. Am. J. Hum. Genet. 93, 1135–1142 10.1016/j.ajhg.2013.10.027 24290375 PMC3853132

[3] Yamada G., Mansouri A., Torres M., Stuart E.T., Blum M., Schultz M. (1995) Targeted mutation of the murine goosecoid gene results in craniofacial defects and neonatal death. Development 121, 2917–2922 7555718 10.1242/dev.121.9.2917

[4] Poirson J., Biquand E., Straub M.L., Cassonnet P., Nominé Y., Jones L. (2017) Mapping the interactome of HPV E6 and E7 oncoproteins with the ubiquitin-proteasome system. FEBS J. 284, 3171–3201 10.1111/febs.14193 28786561

[5] Zou W., Chen X., Shim J.H., Huang Z., Brady N., Hu D. (2011) The E3 ubiquitin ligase Wwp2 regulates craniofacial development through mono-ubiquitylation of Goosecoid. Nat. Cell Biol. 13, 59–65 10.1038/ncb2134 21170031 PMC3059716

[6] Shao R., Liu J., Yan G., Zhang J., Han Y., Guo J. (2016) Cdh1 regulates craniofacial development via APC-dependent ubiquitination and activation of Goosecoid. Cell Res. 26, 699–712 10.1038/cr.2016.51 27126000 PMC4897181

[7] Daneshvar K., Pondick J.V., Kim B.M., Zhou C., York S.R., Macklin J.A. (2016) DIGIT is a conserved long noncoding RNA that regulates GSC expression to control definitive endoderm differentiation of embryonic stem cells. Cell Rep. 17, 353–365 10.1016/j.celrep.2016.09.017 27705785 PMC5120872

[8] Izzi L., Narimatsu M. and Attisano L. (2008) Sumoylation differentially regulates Goosecoid-mediated transcriptional repression. Exp. Cell Res. 314, 1585–1594 10.1016/j.yexcr.2008.01.023 18336814

[9] Latinkic B.V., Umbhauer M., Neal K.A., Lerchner W., Smith J.C. and Cunliffe V. (1997) The Xenopus Brachyury promoter is activated by FGF and low concentrations of activin and suppressed by high concentrations of activin and by paired-type homeodomain proteins. Genes Dev. 11, 3265–3276 9389657 10.1101/gad.11.23.3265 PMC316753

[10] Rivera-Pérez J.A., Mallo M., Gendron-Maguire M., Gridley T. and Behringer R.R. (1995) Goosecoid is not an essential component of the mouse gastrula organizer but is required for craniofacial and rib development. Development 121, 3005–3012 7555726 10.1242/dev.121.9.3005

[11] Boucher D.M., Schäffer M., Deissler K., Moore C.A., Gold J.D., Burdsal C.A. (2000) Goosecoid expression represses Brachyury in embryonic stem cells and affects craniofacial development in chimeric mice. Int. J. Dev. Biol. 44, 279–288 10853824

[12] Tassabehji M., Hammond P., Karmiloff-Smith A., Thompson P., Thorgeirsson S.S., Durkin M.E. (2005) GTF2IRD1 in craniofacial development of humans and mice. Science 310, 1184–1187 10.1126/science.1116142 16293761

[13] Yao J. and Kessler D.S. (2001) Goosecoid promotes head organizer activity by direct repression of Xwnt8 in Spemann’s organizer. Development 128, 2975–2987 11532920 10.1242/dev.128.15.2975

[14] Jin J., Liu J., Chen C., Liu Z., Jiang C., Chu H. (2016) The deubiquitinase USP21 maintains the stemness of mouse embryonic stem cells via stabilization of Nanog. Nat. Commun. 7, 13594 10.1038/ncomms13594 27886188 PMC5133637

[15] Latinkic B. and Smith J. (1999) Goosecoid and mix.1 repress Brachyury expression and are required for head formation in Xenopus. Development 126, 1769–1779 10079237 10.1242/dev.126.8.1769

[16] Nijman S.M., Luna-Vargas M.P., Velds A., Brummelkamp T.R., Dirac A.M., Sixma T.K. (2005) A genomic and functional inventory of deubiquitinating enzymes. Cell 123, 773–786 10.1016/j.cell.2005.11.007 16325574

[17] Smith T.S. and Southan C. (2000) Sequencing, tissue distribution and chromosomal assignment of a novel ubiquitin-specific protease USP23. Biochim. Biophys. Acta 1490, 184–188 10.1016/S0167-4781(99)00233-X 10786635

[18] Gong L., Kamitani T.S. and Yeh E. (2000) Identification of a novel isopeptidase with dual specificity for ubiquitin- and NEDD8-conjugated proteins. J. Biol. Chem. 275, 14212–14216 10.1074/jbc.275.19.14212 10799498

[19] Zhang J., Chen C., Hou X., Gao Y., Lin F., Yang J. (2013) Identification of the E3 deubiquitinase ubiquitin-specific peptidase 21 (USP21) as a positive regulator of the transcription factor GATA3. J. Biol. Chem. 288, 9373–9382 10.1074/jbc.M112.374744 23395819 PMC3611007

[20] Ye Y., Akutsu M., Reyes-Turcu F., Enchev R.I., Wilkinson K.D. and Komander D. (2011) Polyubiquitin binding and cross-reactivity in the USP domain deubiquitinase USP21. EMBO Rep. 12, 350–357 10.1038/embor.2011.17 21399617 PMC3077245

[21] Ristic G., Tsou W.L., Guzi E., Kanack A.J., Scaglione K.M. and Todi S.V. (2016) USP5 is dispensable for mono-ubiquitin maintenance in Drosophila. J. Biol. Chem. 291, 9161–9172 10.1074/jbc.M115.703504 26917723 PMC4861482

[22] Renatus M., Parrado S.G., D’Arcy A., Eidhoff U., Gerhartz B., Hassiepen U. (2006) Structural basis of ubiquitin recognition by the deubiquitinating protease USP2. Structure 14, 1293–1302 10.1016/j.str.2006.06.012 16905103 PMC7126176

[23] Fan Y., Mao R., Yu Y., Liu S., Shi Z., Cheng J. (2014) USP21 negatively regulates antiviral response by acting as a RIG-I deubiquitinase. J. Exp. Med. 211, 313–328 10.1084/jem.20122844 24493797 PMC3920558

[24] Chen Y., Wang L., Jin J, Luan Y., Chen C., Li Y. (2017) p38 inhibition provides anti-DNA virus immunity by regulation of USP21 phosphorylation and STING activation. J. Exp. Med. 214, 991–1010 10.1084/jem.20161387 28254948 PMC5379979

[25] Tao L., Chen C., Song H., Piccioni M., Shi G. and Li B. (2013) Deubiquitination and stabilization of IL-33 by USP21. Int. J. Clin. Exp. Pathol. 7, 4930–4937 PMC4152054 25197364

[26] Xu G., Tan X., Wang H., Sun W., Shi Y., Burlingame S. (2010) Ubiquitin-specific peptidase 21 inhibits tumor necrosis factor alpha-induced nuclear factor kappa B activation via binding to and deubiquitinating receptor-interacting protein 1. J. Biol. Chem. 285, 969–978 10.1074/jbc.M109.042689 19910467 PMC2801298

[27] Kwon S.K., Lee D.H., Kim S.Y., Park J.H., Choi J. and Baek K.H. (2017) Ubiquitin-specific protease 21 regulating the K48-linked polyubiquitination of NANOG. Biochem. Biophys. Res. Commun. 482, 1443–1448 10.1016/j.bbrc.2016.12.055 27956178

[28] Garcíasantisteban I., Bañuelos S. and Rodríguez J.A. (2012) A global survey of CRM1-dependent nuclear export sequences in the human deubiquitinase family. Biochem. J. 441, 209–217 10.1042/BJ20111300 21888622

[29] Okuda H., Ohdan H., Nakayama M., Koseki H., Nakagawa T. and Ito T. (2013) The USP21 short variant (USP21SV) lacking NES, located mostly in the nucleus in vivo, activates transcription by deubiquitylating ubH2A in vitro. PLoS ONE 8, e79813 10.1371/journal.pone.0079813 24278184 PMC3838379

[30] Urbé S., Liu H., Hayes S.D., Heride C., Rigden D.J. and Clague M.J. (2012) Systematic survey of deubiquitinase localization identifies USP21 as a regulator of centrosome- and microtubule-associated functions. Mol. Biol. Cell 23, 1095–1103 10.1091/mbc.e11-08-0668 22298430 PMC3302736

[31] Bouchet B.P. and Akhmanova A. (2017) Microtubules in 3D cell motility. J. Cell Sci. 130, 39–50 10.1242/jcs.189431 28043967

[32] Basiri M.L., Blachon S., Chim Y.C. and Avidor-Reiss T. (2013) Imaging centrosomes in fly testes. J. Vis. Exp. 79, e50938 10.3791/50938 PMC3885179 24084634

[33] Heride C., Rigden D.J., Bertsoulaki E., Cucchi D., De Smaele E., Clague M.J. (2016) The centrosomal deubiquitylase USP21 regulates Gli1 transcriptional activity and stability. J. Cell Sci. 129, 4001–4013 27621083 10.1242/jcs.188516 PMC5117204

[34] Yu J., Huang W.L., Xu Q.G., Z L., S S.H., Z W.P. (2018) Overactivated neddylation pathway in human hepatocellular carcinoma. Cancer Med. 7, 3363–3372 10.1002/cam4.1578 PMC6051160 29846044

[35] Li W., Cui K., Prochownik E.V. and L Y.J. (2018) The deubiquitinase USP21 stabilizes MEK2 to promote tumor growth. Cell Death Dis. 9, 482 29706623 10.1038/s41419-018-0523-z PMC5924753

[36] Chen Y., Zhou B. and Chen D. (2017) USP21 promotes cell proliferation and metastasis through suppressing EZH2 ubiquitination in bladder carcinoma. Oncotargets Ther. 10, 681–689 10.2147/OTT.S124795 28223825 PMC5308592

[37] Peng L., Hu Y., Chen D., Linghu R., Wang Y., Kou X. (2016) Ubiquitin specific protease 21 upregulation in breast cancer promotes cell tumorigenic capability and is associated with the NOD-like receptor signaling pathway. Oncol. Lett. 12, 4531–4537 10.3892/ol.2016.5263 28105162 PMC5228564

[38] Nagasaka A., Shinoda T., Kawaue T., Suzuki M., Nagayama K., Matsumoto T. (2016) Differences in the mechanical properties of the developing cerebral cortical proliferative zone between mice and ferrets at both the tissue and single-cell levels. Front. Cell Dev. Biol. 4, 139 27933293 10.3389/fcell.2016.00139 PMC5122735

[39] Van Delinder V., Adams P.G. and Bachand G.D. (2016) Mechanical splitting of microtubules into protofilament bundles by surface-bound kinesin-1. Sci. Rep. 6, 39408 10.1038/srep39408 28000714 PMC5175155

[40] Jullien J., Vodnala M., Pasque V., Oikawa M., Miyamoto K., Allen G. (2017) Gene resistance to transcriptional reprogramming following nuclear transfer is directly mediated by multiple chromatin-repressive pathways. Mol. Cell 65, 873–884 10.1016/j.molcel.2017.01.030 28257702 PMC5344684

[41] Rivkin E., Almeida S.M., Ceccarelli D.F., Juang Y.C., MacLean T.A., Srikumar T. (2013) The linear ubiquitin-specific deubiquitinase gumby regulates angiogenesis. Nature 498, 318–324 10.1038/nature12296 23708998 PMC4931916

[42] Pannu J., Belle J.I., Förster M., Duerr C.U., Shen S., Kane L. (2015) Ubiquitin specific protease 21 is dispensable for normal development, hematopoiesis and lymphocyte differentiation. PLoS ONE 10, e0117304 10.1371/journal.pone.0117304 25680095 PMC4332479

