# Supplementary material for: USP21 modulates Goosecoid function through deubiquitination
Source: Biosci Rep. 2019 Jul 10;39(7):BSR20182148. doi: 10.1042/BSR20182148 (PMC6620385; doi:10.1042/BSR20182148)
Supplement: Supplementary file 1 [file bsr20182148_Supp1.pdf]

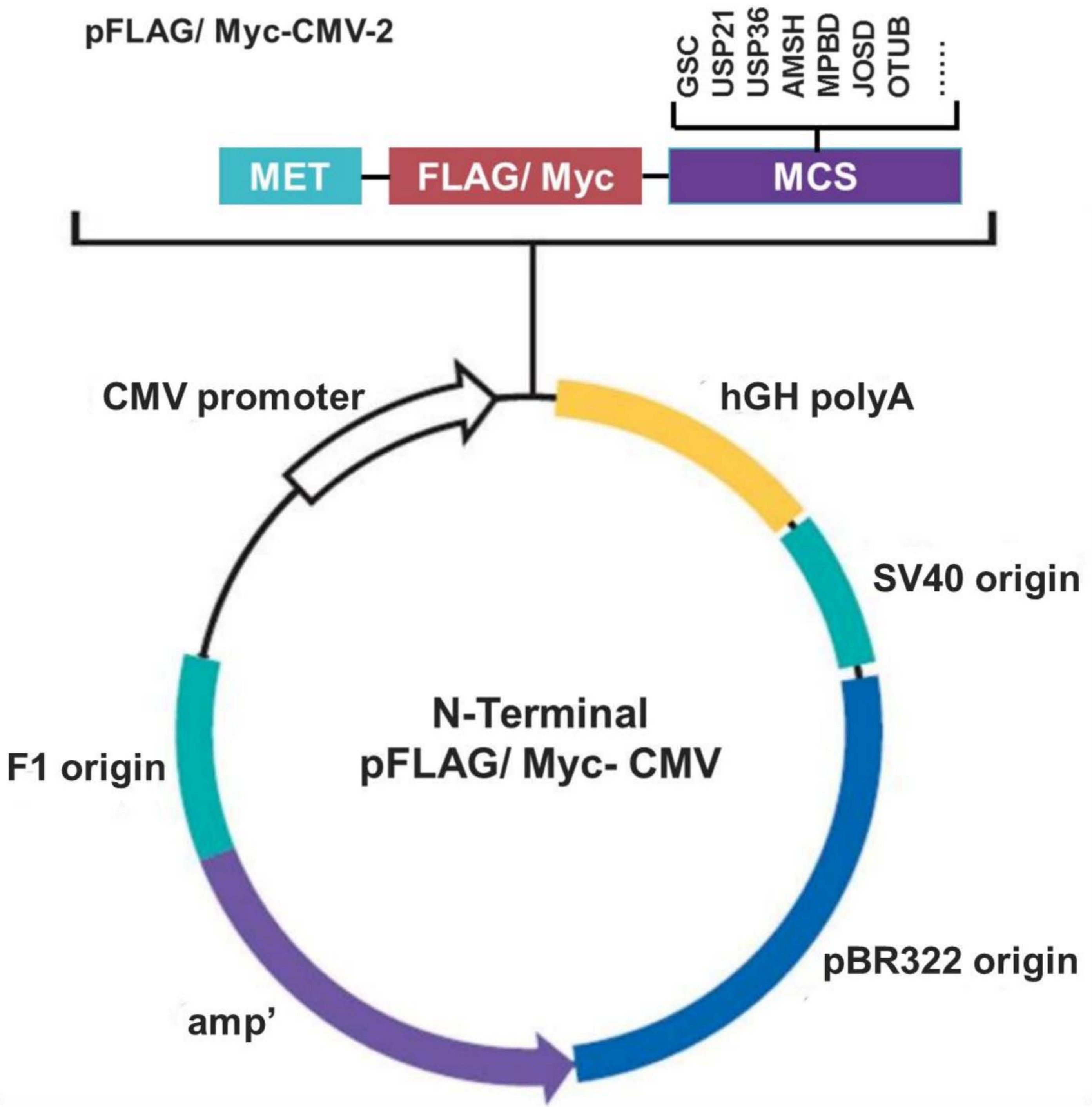
pFLAG/ Myc-CMV-2
GSC
USP21
USP36
AMSH
MPBD
JOSD
OTUB
......
MET
FLAG/ Myc
MCS
CMV promoter
hGH polyA
SV40 origin
N-Terminal
pFLAG/ Myc- CMV
F1 origin
pBR322 origin
amp'

Table S1. Information about the Antibodies.

	Antibodies's name	Brand	Product Number
1	Monoclonal Anti-c-Myc antibody produced in mouse - clone 9E10	Sigma	M4439
2	Monoclonal ANTI-FLAG® M2 antibody produced in mouse - clone M2	Sigma	F3165
3	Monoclonal Anti-HA, antibody produced in mouse	Sigma	H9658
4	Monoclonal Anti-HSP90 antibody produced in mouse	Sigma	SAB1305541
5	Anti-Collagen Type II Antibody	Sigma	MAB8887
6	Anti-Mouse IgG (whole molecule)–Peroxidase antibody produced in rabbit	Sigma	A9044
